# Case report: A giant lung leiomyosarcoma: From an accurate diagnosis to a successful surgery. A rare case and brief literature review

**DOI:** 10.3389/fsurg.2022.975982

**Published:** 2022-09-01

**Authors:** Elisa Sicolo, Maria Giovanna Mastromarino, Giacomo Rabazzi, Diana Bacchin, Greta Ali, Vittorio Aprile, Marco Lucchi

**Affiliations:** ^1^ Thoracic Surgery Division, Cardiac, Vascular and Thoracic Department, University Hospital of Pisa, Pisa, Italy; ^2^ Unit of Pathological Anatomy, University Hospital of Pisa, Pisa, Italy

**Keywords:** primary lung leiomyosarcoma, lung cancer, thoracic surgery, sterno-thoracotomy, pneumonectomy, transcatheter arterial embolization

## Abstract

Primary pulmonary leiomyosarcomas (PPLs) are incredibly rare tumors, characterized by an often-aggressive clinical behavior. Diagnosis is frequently incidental. Whenever present, symptoms are nonspecific, thus PPLs are usually misdiagnosed as other more common respiratory diseases or lung cancer subtypes. Surgery is the best treatment choice and in the therapeutic strategy, timeliness and radicality are of major importance. We report the case of a huge left PPL, successfully treated with salvage surgery after a careful multidisciplinary preoperative assessment, which permitted a prompt diagnostic path and surgery.

## Introduction

Primary pulmonary leiomyosarcoma (PPL) is an exceedingly rare and clinically aggressive tumor, accounting for less than 0.5% of all lung malignancies (1). PPLs may originate from the smooth muscle cells of the lung interstitium, bronchial tree, or blood vessel walls (2). Diagnosis is often incidentally and based on radiological findings at chest x-ray or chest computed tomography (CT)-scan. Due to their rarity and nonspecific symptoms, PPLs are frequently overlooked and misdiagnosed as other more common respiratory diseases or lung cancer subtypes. PPLs typically show a lethal clinical attitude, especially in case of diagnostic delay. The treatment is aimed at preserving patient function and restoring quality of life. Surgery represents the mainstay of potentially curative therapy of localized PPL, offering patients an improved prognosis (3).

We report a case of a left huge PPL successfully radical resected, initially misdiagnosed as massive pleural effusion and rapidly impairing the patient's respiratory function. Symptoms onset was characterized by shortness of breath and orthopnoea, which required the hospitalization of the patient. CT scan revealed the presence of a huge mass in the left hemithorax.

This report emphasizes the paramount importance of an accurate diagnosis and a proper multidisciplinary perioperative assessment to favorably manage rare tumors as PPLs.

## Case description

A 71-year-old woman was admitted to the emergency department of our tertiary care center for worsening shortness of breath and orthopnoea. She was a never-smoker and her past medical history was significant for asthma, diabetes mellitus, congenital solitary kidney, dyslipidaemia, and previous surgical treatment of Arnold-Chiari malformation in childhood.

**Figure 1:**
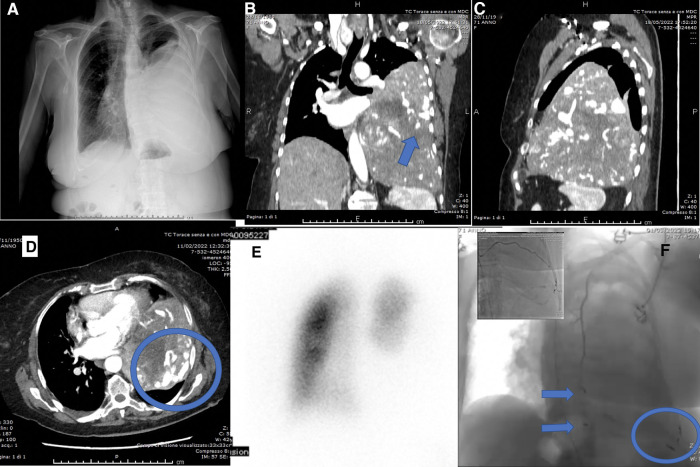
(**A**) Preoperative chest x-ray: left side uniformly white with mediastinal shift; (**B**) preoperative chest CT showing the mass with abnormal angiogenesis (arrow), coronal scan; (**C**) preoperative chest CT, sagittal scan; (**D**) preoperative chest CT showing the abnormal tumor angiogenesis (round), axial scan; (**E**) lung perfusion scintigraphy; (**F**) transcatheter arterial embolization (TAE) of X, XI, XII left intercostal arteries (round), left superior phrenic artery (inferior arrow), left internal mammary artery (superior arrow).

Physical examination revealed dullness to percussion, decreased tactile fremitus, and asymmetrical chest expansion, with diminished expansion on the left side. Chest x-ray showed a left side uniformly white with mediastinal shift, thus a diagnosis of massive left pleural effusion was suspected (Figure 1A). Considering the quickly progressive dyspnea, an emergency thoracentesis was attempted, but it was unsuccessful.

A chest–abdomen CT scan was taken to rule out the hypothesis of a life-threatening pleural effusion. It demonstrated a huge enhancing left pleural mass, with a diameter of 18.4 × 16.0 × 11.2 cm, causing compression atelectasis of the left lung and mediastinal shift, with abnormal tumor neovascularization (Figures 1B–D). As a collateral finding, a solitary left gluteal lesion of a few millimeters was also detected, presenting a contrast enhancement similar to the chest mass, but it was extremely small to be biopsied.

Because of the abnormal tumor angiogenesis, a CT-guided needle biopsy of the mass, aimed to obtain a tumor histology, was not feasible due to the high risk of bleeding, as well as bronchial washing, brushing, or biopsy.

To assess the best therapeutic approach, the case was discussed on a lung cancer dedicated multidisciplinary board (MDB). Considering the poor role both of radiotherapy and chemotherapy given the huge tumor size and rapidly worsening orthopnoea, the patient was a candidate to salvage surgery and subsequent follow-up of the left gluteal lesion of unknown origin.

The preoperative assessment included Lung Perfusion Scintigraphy showing complete hypoperfusion of the left lung related to the mass (left lung 12.8%, right lung 87.2%) (Figure 1E). Cardiological evaluation with echocardiography and coronary angiography found no significant alterations. The tumor staging was completed with Brain CT, negative for metastases.

To reduce the risk of intraoperative bleeding, a preoperative transcatheter arterial embolization (TAE) of the mass was performed. Transcatheter angiography through the right femoral artery revealed vascular supply from X, XI, XII left intercostal arteries, left superior phrenic artery, left internal mammary artery (Figure 1F) and TAE was carried out with coils.

Due to the uncertain relationship between the mass and the pulmonary left hilar structures, and the deep narrow surgical field related to the huge size of the neoplasm, the operating room was equipped to perform extracorporeal circulation (CEC) to sustain systemic blood flows during eventually dissection involving the heart or great vessels.

**Figure 2:**
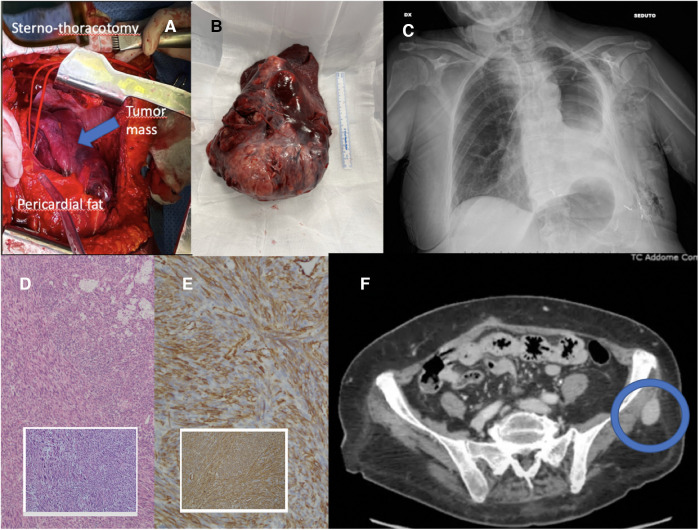
(**A**) Intraoperative view of hemiclamshell incision; (**B**) left lung en-bloc with mass; (**C**) postoperative chest x-ray; (**D**) hematoxylin-eosin, (10×, 4×) leiomyosarcoma of the lung; (**E**) immunohistochemistry, smooth muscle actin and muscle-specific actin (10×); (**F**) CT scan showing left gluteal metastasis.

The patient underwent a sterno-thoracotomy (hemiclamshell incision) at the fifth left intercostal space (Figure 2A). Intraoperative exploration revealed a giant left intrathoracic mass, almost completely occupying the pleural cavity, infiltrating the left lung, characterized by hypervascularity and diaphragmatic adhesions. A pericardial fat pad resulted infiltrated by the neoplasm and then resected. The pericardium was opened, with evacuation of modest pericardial effusion. Pulmonary hilar structures appeared not infiltrated by the mass. An intrapericardial left pneumonectomy with radical en-bloc mass resection was performed (Figure 2B). Additionally, diaphragmatic adhesions were released. Surgery was carried out without CEC. The surgical procedure is briefly shown in Supplementary Video 1.

The patient was admitted to the intensive care unit (ICU) for postoperative continuous monitoring. Extubation was performed on the operation day without complications. On the third postoperative day (POD), the patient was discharged from ICU and returned to the ward, where the chest tube was removed (Figure 2C). The postoperative course was uneventful. The patient was discharged on the 15th POD and addressed to postacute respiratory and physical rehabilitation.

The surgical pathology report revealed a large malignant mesenchymal tumor (17 × 15 × 9 cm) characterized by spindle cells with significantly increased areas of mitotic activity (Figure 2D). The average mitotic rate was 21 mitotic figures per 10 high-powered fields. Areas of necrosis and hemorrhage were also identified (20% of the mass). Bronchial and vascular resection margins were free from tumor infiltration. Immunohistochemistry (IHC) showed malignant spindle cell positivity for Actin 1A4, Actin HHF-35, Caldesmon, and Desmin (Figure 2E). Malignant spindle cells tested negative for Pan-cytokeratin (CK), CK-CAM5.2, STAT6, CD34, S100, HMB45, and SOX10. The MIB-1 proliferation index was noted as 65%. One hilar lymph node out of seven resulted positive for metastatic disease.

Overall morphologic features favored a high-grade sarcoma with evidence of smooth muscle differentiation; therefore a diagnosis of PPL, grade 3–FNCLCC, was rendered.

**Figure 3:**
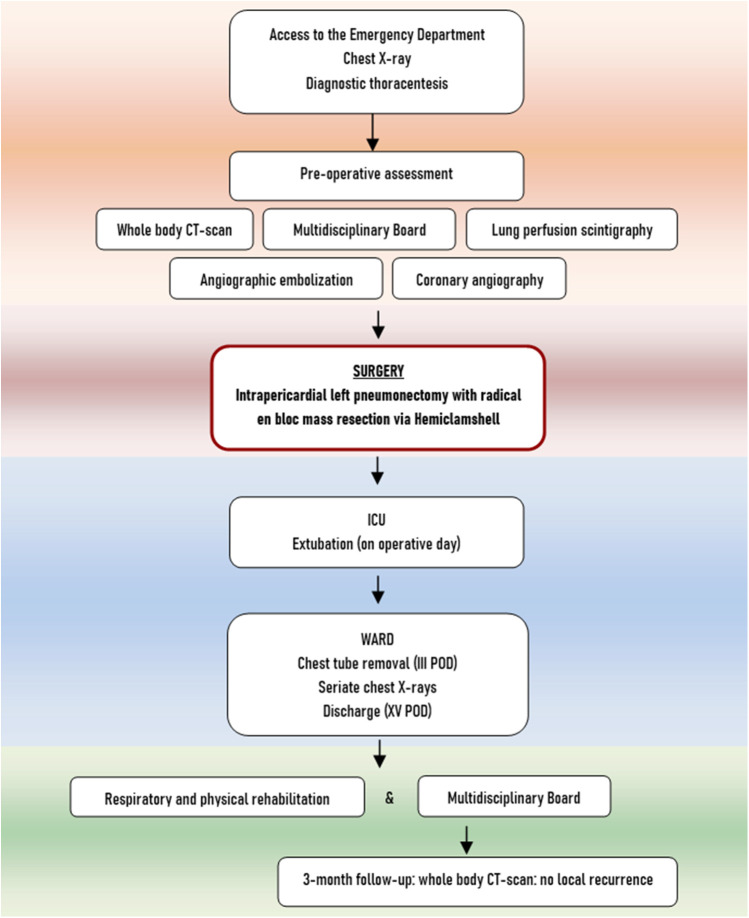
Table showcasing of care timeline.

After a 3-month follow-up, the patient had favorably concluded respiratory and physical rehabilitation and presented with good performance status. The whole body CT scan showed common post-pneumonectomy features, with no evidence of local recurrence. The left enhancing gluteal lesion presented increasing in size (diameter of 28 × 20 mm), therefore an ultrasound-guided needle biopsy was performed, but it revealed non-diagnostic (Figure 2F). However, considering the imaging features highly suggestive of metastasis, the patient was then a candidate for surgical excision of the left gluteal lesion (the pathology report confirming the diagnosis as metastatic lesion and surgical specimen picture are provided in the Supplementary Material). The care timeline is shown in Figure 3.

## Discussion

PPLs are very rare malignant tumors, accounting for less than 0.5% of all lung malignancies and representing the most common histologic subtypes of sarcomas that occur in the lung (3, 4). PPLs usually affect people over 60 years of age, with a prevalence in men, although some cases have been described in younger populations and children (2, 5). PPLs are clinically aggressive tumors and often lethal, due to rapid growth (5). Metastases are uncommon and typically occur late in disease progression (6). Considering their extreme and the fact that leiomyosarcomas can arise from any location, especially in female patients, it is essential to distinguish a primary lung tumor from metastases. An accurate complete physical examination should always be performed to rule out the presence of a primary tumor elsewhere in the body, focusing on the uterus, gastrointestinal tract, and soft tissue (3). Most PPLs arise from tissues surrounding the hilum, as seen in the patient in the present report. The origin is presumably from the smooth muscle cells of the lung interstitium, bronchial tree, or blood vessel walls and they can be classified as intraluminal, intrapulmonary, and pulmonary vascular (2, 3, 7). The intrapulmonary type is the most common, as in this report.

PPLs are often asymptomatic on presentation and incidentally founded on routine chest radiography. Symptoms, when present, are similar to other primary pulmonary malignancies such as cough, hemoptysis, dyspnea, asthenia, chest and back pain, and weight loss. This type of tumor is frequently undetected or misdiagnosed, due to the nonspecific clinical presentation as other more common lung diseases, as in the present case. A first diagnosis of life-threatening pleural effusion was indeed supposed (8). PPLs should be suspected when a certain radiological pattern is associated with no smoking history. A solitary oval or round, well-circumscribed, calcified and heterogeneous nodule with smooth margins or large necrotic masses on chest imaging could be suggestive of a PPL, along with other types of lung cancer (4, 5). Therefore, a tumor biopsy with proper IHC tests is required to provide a definitive diagnosis. Moreover, bronchial washing or brushing could be useful (3). Nonetheless, in our case the mass presented with abnormal tumor angiogenesis, making biopsy not safe for the high risk of bleeding. Consequently, the patient was a candidate for upfront salvage surgery, considering the symptomatic onset of the disease and rapidly worsening dyspnea requiring oxygen support therapy. Currently, surgery represents the gold-standard treatment of localized PPL, and it is generally considered “curative” if completeness of resection is achieved, allowing patients improved prognosis, with a 5-year survival rate close to 50% (7). The goal of the treatment is to obtain both local and systemic control of the sarcoma while preserving function and quality of life (7). Typically, patients undergo lung lobectomy, pneumonectomy, or bronchial sleeve resection. Patients with large PPLs may receive preoperative radiation treatment in hopes of decreasing the size of the mass before surgery. Adjuvant radiotherapy, chemotherapy, or both may be indicated in case of unresectable mass, incomplete resection, or high-grade tumors (3, 7). Surgery remains the first-choice treatment even in advanced PPLs presenting with distant metastasis, if a radical local control of the metastatic site can be achieved (9). Although our patient presented with a gluteal lesion suspicious of metastasis at diagnosis, salvage surgery was indicated as a priority over radiotherapy or chemotherapy, considering the huge size of the mass and the rapidly worsening shortness of breath. Surgical resection would have been the only chance for effective and definitive treatment. Given the very small size and the solitary nature of the gluteal nodule, it was initiated for follow-up.

A sterno-thoracotomy (Hemiclamshell incision) was performed because of the huge size of the PPL. As previously described (9), a wide surgical field can allow better control of the hilum structures and more direct access to the chest “blind zones”, such as heart and great vessels, considering also the eventual needing of CEC. Similarly, Porrello et al. performed a right pneumonectomy *via* hemiclamshell incision for a giant mediastinal leiomyosarcoma. The mass was located on the right mediastinum encasing the right main pulmonary artery and infiltrating the main right bronchus and pericardium. Sterno-thoracotomy provided an adequate exposure of the chest and allowed a radical and safe surgical resection of the tumor en-bloc with the lung, pleura, pericardium, and diaphragm (10).

Preoperative TAE proved to be of paramount importance to prevent intraoperative bleeding. According to the literature, TAE can be considered a successful and effective technique in preventing intra- and postoperative blood loss, as demonstrated by Yuza and colleagues in surgical excision of giant pelvic solitary fibrous tumors (11). TAE has proved to be a powerful weapon for avoiding massive bleeding also in oncological orthopedic surgery, such as in resection and reconstruction of long bone hypervascular tumors (12). In the management of this case, the whole careful preoperative assessment allowed surgery to be successfully carried out and feasible without CEC, as well as postoperative course was uneventful.

PPLs prognostic factors consist of tumor size, extent of bronchial invasion, grading of malignancy as well as pathological differentiation, stage, primary site of tumor, lymph node, and distant metastases (2, 7).

Metastases are uncommon and typically occur late in the disease clinical course, therefore the early diagnosis and the complete surgical resection of PPLs may significantly contribute to increased patient survival (5, 6).

We searched PubMed, Embase, and Web of Science (up to and including May 2022) for published articles using the search terms “primary pulmonary leiomyosarcoma”; almost 300 patients with PPLs were identified.

The first case of PPL was described by Davidsohn in 1903 (5). Most of the evidence consists of a single case report or small series, due to the rarity of PPLs. Qin et al. carried out a retrospective analysis of patients affected by PPL and registered in the Surveillance, Epidemiology and End Results (SEER) Database, finding a total of 231 cases from 1973 to 2013 (2). Similarly, Sardenberg and colleagues reached about 300 cases in their research involving more backward years, starting from 1903 to 2006 (4). The largest population cohort study by Qin and co-authors confirmed surgery as the mainstay treatment in resectable disease, even if it has been technically feasible in about 60% of patients (2). Surgery significantly improved long-term outcomes and whenever an early complete resection was achieved, 5-year survival exceeded 50%, and there have been reports of patient survival for 20 years post resection (3). Accordingly, Shaw et al. reported 20-year survival of 63%, with no disease recurrence (13). The population-based study also demonstrated that radiotherapy and chemotherapy did not benefit PPL patients in overall survival (2). Conversely, these treatment regimens are recommended for cases of incomplete resection, unresectable tumors, and patients with increased histological malignancy (2).

Our case presents some remarkable distinctive features, first of all, the huge size of the mass, not infiltrating any vital structure. Giant resectable PPLs are rarely described, given their local aggressive behavior with the involvement of neighboring organs. Evidence has shown that among hundreds of patients who underwent surgery, the vast majority received lobectomy, bi-lobectomy, or partial resection, while only in a small minority of cases pneumonectomy was performed, as in the present rare case. Second, the mass presented with the very peculiar characteristic of abnormal, life-threatening angiogenesis. To our knowledge, this is the first case of giant PPL reporting the use of a preoperative TAE to effectively prevent the risk of intraoperative bleeding. The main limit of this report is represented by the lack of preoperative diagnosis. Nevertheless, the abnormal tumor neovascularization did not allow to safely perform a percutaneous biopsy, as well as surgery was undeferrable given the rapid worsening of the patient's conditions, suggestive of clinical deterioration.

## Conclusion

PPL is an exceedingly rare and clinically aggressive tumor, characterized by rapid growth. PPLs often remain undetected or misdiagnosed, due to nonspecific symptoms as other more common lung diseases. Surgeons and pathologists should be aware of this rare lesion and keep it in their differential diagnosis with other pulmonary tumors, although it may be challenging due to the lack of specific manifestations. Despite their aggressive and often lethal clinical behavior, surgery remains the pivotal treatment for PPLs to obtain local control. Even in the case of huge mass, surgical resection should always be attempted to offer a chance of cure to these patients with ominous prognoses.

Our case suggests that in a high volume, experienced thoracic center, careful staging, and a proper multidisciplinary perioperative assessment can achieve the goal of successful radical surgery for PPLs.

## Data Availability

The original contributions presented in the study are included in the article/Supplementary Material, further inquiries can be directed to the corresponding author/s.

## Supplementary material

Supplementary Video 1 Surgical procedure and postoperative CT.
