# Supplementary material for: Case report: A giant lung leiomyosarcoma: From an accurate diagnosis to a successful surgery. A rare case and brief literature review
Source: Front Surg. 2022 Sep 1;9:975982. doi: 10.3389/fsurg.2022.975982 (PMC9475251; doi:10.3389/fsurg.2022.975982)
Supplement: Supplementary file 2 [file Data_Sheet_1.pdf]

Pathology report of left gluteal metastasis

The surgical pathology report revealed a subcutaneous metastasis of pulmonary leiomyosarcoma (3.8 x 3.2 x 2.4 cm). Resection margins were free from tumour infiltration. Immunohistochemistry (IHC) showed malignant spindle cells positivity for Actin 1A4, Actin HHF-35 and Caldesmon.

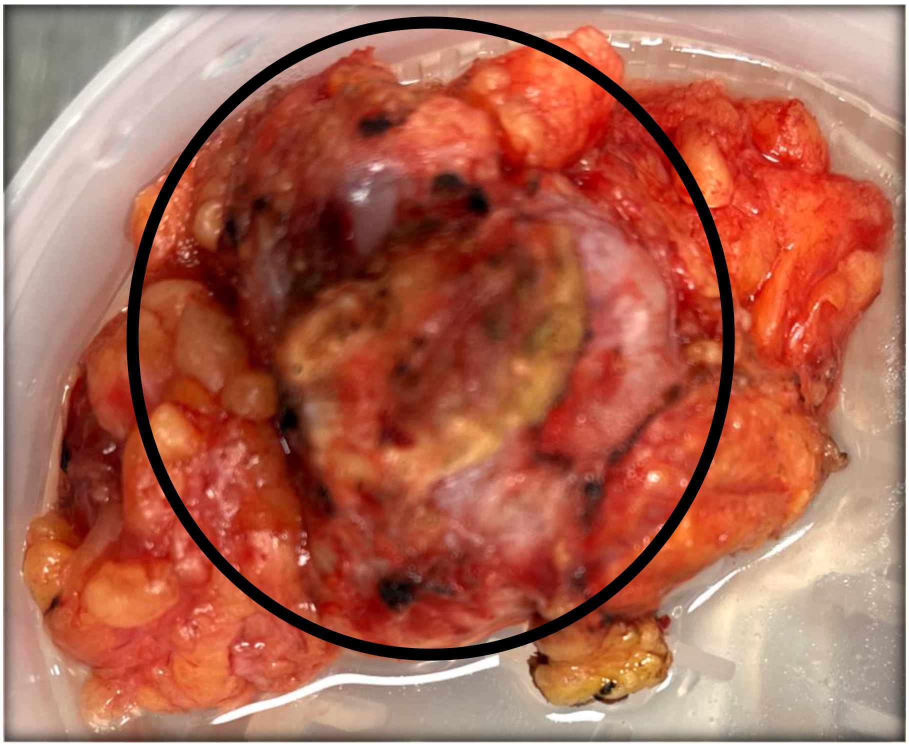

Surgical specimen of subcutaneous left gluteal metastasis.
